# Voltage control of magnetic order in RKKY coupled multilayers

**DOI:** 10.1126/sciadv.add0548

**Published:** 2023-01-04

**Authors:** Alexander E. Kossak, Mantao Huang, Pooja Reddy, Daniel Wolf, Geoffrey S. D. Beach

**Affiliations:** ^1^ Department of Materials Science and Engineering, Massachusetts Institute of Technology, Cambridge, MA 02139, USA.; ^2^ Leibniz IFW Dresden Helmholtzstrasse 20, Dresden 01069, Germany.

## Abstract

In the field of antiferromagnetic (AFM) spintronics, there is a substantial effort present to make AFMs viable active components for efficient and fast devices. Typically, this is done by manipulating the AFM Néel vector. Here, we establish a method of enabling AFM active components by directly controlling the magnetic order. We show that magneto-ionic gating of hydrogen enables dynamic control of the Ruderman-Kittel-Kasuya-Yosida (RKKY) interaction in solid-state synthetic AFM multilayer devices. Using a gate voltage, we tune the RKKY interaction to drive continuous transitions from AFM to FM and vice versa. The switching is submillisecond at room temperature and fully reversible. We validate the utility of this method by demonstrating that magneto-ionic gating of the RKKY interaction allows for 180° field-free deterministic switching. This dynamic method of controlling a fundamental exchange interaction can engender the manipulation of a broader array of spin textures, e.g., chiral domain walls and skyrmions.

## Teaser

Voltage-loading of hydrogen modifies magnetic interactions in multilayered films to dynamically control the net magnetic order.

## INTRODUCTION

The existence of magnetic order emerges from a competition of several interactions foremost being the exchange interaction. The energy difference between neighboring spins can be described by the Heisenberg model, $H_{\mathrm{ex}}=-2\sum_{i<j}J_{\mathrm{ij}}S_{i}\cdot S_{j}$, where the sign of the exchange constant *J*_ij_ determines the primary classification of the magnetic material, i.e., ferromagnetic (FM) or antiferromagnetic (AFM). When magnetic layers are stacked into a heterostructure, several other types of exchange can occur, e.g., the interfacial Dzyaloshinskii-Moriya interaction (*1*, *2*) and the Ruderman-Kittel-Kasuya-Yosida (RKKY) interaction (*3*–*5*). By using these nonlocal interactions with particular materials and geometries, magnetic order can be engineered. The fabrication of synthetic FMs and AFMs is achieved by controlling the RKKY interaction via the thickness and material of the magnetic and nonmagnetic layers (*6*). Unfortunately, the nature of the magnetic order is set during synthesis, and the growth of additional heterostructures is required to tune it. Tuning the exchange coupling would provide substantial opportunities in the field of AFM spintronics (*7*–*11*). It has been shown that tailoring the exchange coupling can allow for higher density and velocity domain walls (*12*, *13*), where the capacity is limited by dipolar coupling from fringing magnetic fields (*14*), the stabilization of skyrmions (*15*, *16*), and the mitigation of the skyrmion Hall effect (*17*–*19*). AFMs have traditionally been used as passive components, e.g., biasing layers in magnetic tunnel junctions, while FMs as active components, e.g., bits in magnetic storage media. However, there is a considerable desire to make AFMs active components, e.g., by manipulating their AFM order parameter, due to their ultrafast dynamics, the absence of stray fields, and stability against external magnetic fields (*10*, *20*). Synthetic AFMs, while not intrinsically compensated, can be engineered to share many of the same advantages as crystalline AFMs. By controlling the relative thickness of the constituent FM layers, synthetic AFMs can have zero stray fields (*21*), thereby limiting cross-talk between bits and increasing bit density (*7*). While the exchange interaction in synthetic AFMs is much weaker than in crystalline AFMs and, therefore, more susceptible to external fields, synthetic AFMs can still be engineered to have stable AFM coupling up to several tesla (*7*).

Here, we show that a gate voltage can induce ionic infiltration into a magnetic heterostructure allowing for solid-state, dynamic control of this fundamental exchange interaction and drive continuous transitions from one magnetic class to another. We establish a method of enabling AFM active components not by manipulating the AFM order parameter but by switching between AFM and FM order dynamically. To demonstrate the broad utility of this platform, we engineered a heterostructure that allows for solid-state, voltage-controlled, reversible, 180° field-free switching based on materials already used in magnetic tunnel junction and spin-transfer torque magnetoresistive random-access memory devices (*22*). We show that the RKKY coupling can be controlled by using a small applied voltage to pump hydrogen (H) from the ambient moisture into the magnetic interlayer. The injection of H causes a change in both the amplitude and phase of the decaying oscillatory RKKY coupling. This leads to a change of up to 800 Oe in the exchange field and, near the zero crossings, allows the RKKY coupling to switch from FM to AFM and vice versa. The switching is fully reversible, cyclable, and submillisecond. In the field of spintronic devices, this adds an alternative, low-power, voltage-controlled approach to magnetic switching, and, in the field of magneto-ionics, this adds a new capability to the existing toolkit of voltage-controlled perpendicular magnetic anisotropy (PMA) (*23*–*26*), exchange bias (*27*–*29*), interlayer exchange (*30*, *31*), and interfacial Dzyaloshinskii-Moriya interaction (*32*).

## RESULTS

### Voltage-controlled RKKY coupling

**Fig. 1.**
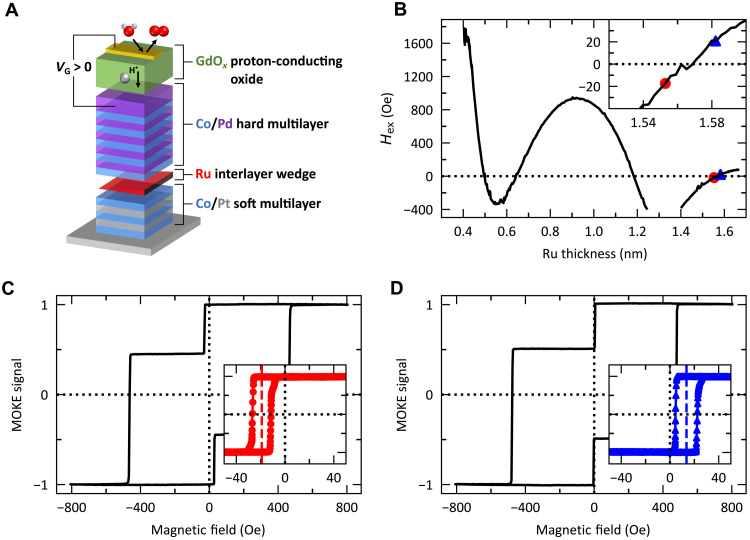
RKKY exchange field coupling as a function of ruthenium interlayer thickness. (**A**) Illustrative schematic of the multilayer heterostructure made of Ta(3)/Pt(2.5)/[Co(0.27)/Pt(1.2)]_2_/Co(0.27)/Ru(0.4–1.7)/Co(0.3)/[Pd(1.2)/Co(0.3)]_5_/Pd(6)/GdO*_x_*(26)/Au(8). (**B**) Exchange field exerted on the soft layer (Co/Pt multilayer) using the minor loop as a function of ruthenium interlayer thickness. The red circle and the blue triangle markers correspond to the regions where the RKKY coupling is FM and AFM, respectively (**C** and **D**). Exemplary major hysteresis loops with the minor hysteresis loop in the inset corresponding to the region indicated by the red circle and blue triangle markers in (**B**), collected via polar MOKE magnetometry.

Our heterostructure is Ta(3)/Pt(2.5)/[Co(0.27)/Pt(1.2)]_2_/Co(0.27)/Ru(0.4–1.7)/Co(0.3)/[Pd(1.2)/Co(0.3)]_5_/Pd(6)/GdO*_x_*(26)/Au(8) (nominal layer thicknesses in nanometers) and was deposited by magnetron sputtering at room temperature on silicon substrates and schematically shown in Fig. 1A. The heterostructure consists of two FM/heavy-metal multilayers separated by a ruthenium (Ru) interlayer wedge. The bottom Co/Pt multilayer acts as the soft, free layer, and the top Co/Pd multilayer acts as the hard, fixed layer. The separating Ru layer generates RKKY coupling and was grown as a thickness wedge yielding a variation in the sign and strength of the RKKY coupling along the sample laterally, shown in Fig. 1B (*6*, *33*, *34*). Cross-sectional high-resolution transmission electron microscopy (HR-TEM) and elemental mapping at the thickest region of the Ru wedge show well-defined Ru interfaces (Fig. S1, A and B). We determined the RKKY coupling strength by the exchange field, *H*_ex_, using polar magneto-optical Kerr effect (MOKE) magnetometry. *H*_ex_ is directly proportional to the RKKY interlayer exchange coupling constant *J*_0_ (*35*).

For symmetric RKKY coupled layers, it is not possible to measure the strength of the FM RKKY coupling using MOKE microscopy because the coercive switching field of both layers is equal, i.e., they will switch simultaneously. However, when the layers have different coercivities, as chosen here, it becomes possible to measure the FM RKKY exchange field ($H_{\mathrm{ex}}^{\mathrm{FM}}$) exerted on the soft layer by collecting minor hysteresis loops. When $H_{c}^{\mathrm{Soft}}+H_{\mathrm{ex}}^{\mathrm{FM}}$ is less (greater) than $H_{c}^{\mathrm{Hard}}-H_{\mathrm{ex}}^{\mathrm{FM}}$, the two layers will switch independently (together) giving a split (single) hysteresis loop. For this reason, despite a sign change of *H*_ex_ and, therefore, sign change of the RKKY coupling, both major loops in Fig. 1 (C and D) have two switching events per sweep direction. Here, the sign of the RKKY coupling is determined using the minor loop and shows FM coupling when *H*_ex_ < 0 (Fig. 1C, inset) and AFM coupling when *H*_ex_ > 0 (Fig. 1D, inset).

**Fig. 2.**
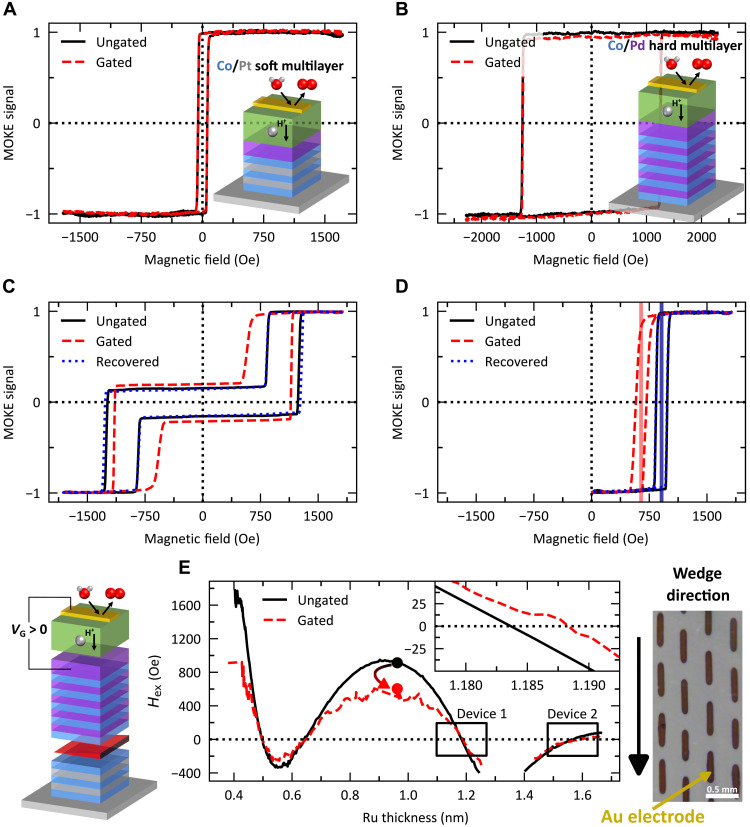
Magneto-ionic gating of the individual multilayers and along the ruthenium wedge. Magneto-ionic gating at +5 V for 5 min for (**A**) Ta(3)/Pt(2.5)/[Co(0.27)/Pt(1.2)]_2_/Co(0.27)/Ru(1)/Pd(6)/GdO*_x_*(26)/Au(8), (**B**) Ta(3)/Pt(4)/Co(0.3)/[Pd(1.2)/Co(0.3)]_5_/Pd(6)/GdO*_x_*(26)/Au(8), and (**C** and **D**) Ta(3)/Pt(2.5)/[Co(0.27)/Pt(1.2)]_2_/Co(0.27)/Ru(0.4–1.7)/Co(0.3)/[Pd(1.2)/Co(0.3)]_5_/Pd(6)/GdO*_x_*(26)/Au(8) (nominal thickness in nanometers). Both the major (**C**) and minor (**D**) hysteresis loops of the multilayer heterostructure are given for the ungated, gated, and recovered states in the region corresponding to the markers in (**E**). (**E**) Exchange field exerted on the soft layer (Co/Pt multilayer) using the minor loop as a function of ruthenium interlayer thickness while magneto-ionically gating at +4 V; the ungated data (black) are given for comparison with circular markers indicating the device in (**C**) and (**D**). An optical micrograph of the electrode layout is shown where the long direction of the electrode is parallel to the thickness gradient of the Ru interlayer.

The Co/Pt multilayer was chosen for its interfacial PMA, which can be tuned by the relative Co and Pt thicknesses (*36*). Moreover, Pt has a relatively low permeability to H (*37*), which ensures that the previously demonstrated magneto-ionic gating of PMA will be minimized (*24*). Figure 2 (A and B) shows hysteresis loops of the individual multilayers before and after gating (*V*_g_ = +5 V for 5 min), both demonstrating robust PMA even in the gated state. Pd was chosen for the top multilayer, due to its high H permeability (*37*), which allows H to reach the Ru interlayer. Last, the Au top gate allows for electrical contact in addition to catalyzing water dissociation from the ambient humidity under *V*_g_ > 0 (*38*). It is this dissociation that generates the proton that transfers through the GdO*_x_* proton–conducting oxide into the magnetic heterostructure where it combines with an e^−^ to yield a charge-neutral species (*39*).

Since PMA is maintained upon gating in both multilayers, the effects of gating on the RKKY coupling can be readily discerned. Applying *V*_g_ = +4 V for 30 s to the RKKY stack, we observed both a change in the major and minor hysteresis loops, shown in Fig. 2 (C and D), respectively. *H*_ex_ > 0 in the minor loop denotes AFM coupling between the two multilayers. Under *V*_g_ application, *H*_ex_ decreases, indicating a decrease in the RKKY strength. Under *V*_g_ = −1 V for 30 s, *H*_ex_ is fully recovered, demonstrating a fully reversible process. By gating several electrodes along the Ru gradient direction, the *V*_g_-induced change in *H*_ex_ was measured as a function of Ru thickness, shown in Fig. 2E. Given that H must diffuse through a much thicker film than previously demonstrated (*24*, *25*, *28*, *39*–*41*), we confirmed its unique potential to manipulate buried layers with H modulation of a similarly buried GdCo layer. We fabricated the heterostructure Ta(3)/Pt(4)/GdCo(8)/Ru(2)/Pd(13.8)/GdO*_x_*(26)/Au(8) (nominal layer thicknesses in nanometers), where the GdCo was Gd-rich and had PMA. Under *V*_g_ = +2 V for 30 s, an inversion of the MOKE polarity and a change in the coercivity take place, indicating H modulation of the dominant sublattice of the GdCo (*41*), shown in fig. S2. Under *V*_g_ = −1 V for 30 s, the original state is fully recovered, indicating that H can diffuse through several nanometers of metal layers and be subsequently removed.

Injection of H into the heterostructure produces two prominent changes in the oscillatory RKKY coupling. First, there is a substantial decrease in the amplitude of the exchange coupling strength. Second, there is a small phase shift, shown in the inset of Fig. 2E. Similar observations were made in theoretical work concerning the influence of an electric field on the RKKY coupling of magnetic impurities (*42*, *43*). The amplitude variation was attributed to a change in the electron density, hence, a change in the RKKY interaction given its reliance on conduction electrons. The phase shift originates from a change in the kinetic energy of the conduction electrons, causing a change in the wavelength of the oscillations. In our system, the change in electronic structure is presumed to arise from the solid-state injection and expulsion of H rather than an electric field. Previous demonstrations of gaseous H modulation of the exchange coupling in Fe/Nb (*44*) and Fe/V (*45*) superlattices determined that the change in RKKY coupling came from H-induced modification of the electronic structure and, therefore, the Fermi surface rather than the lattice constant of the interlayer and, therefore, the interlayer thickness. In our demonstration, we can exclude a trivial thickness-induced origin of the phase shift since an H-induced increase in the lattice constant and, therefore, the film thickness would generate a phase shift to the left, whereas we experimentally observe a phase shift to the right. Explicitly, this would cause a decrease in *H*_ex_ for the device located within the inset of Fig. 2E rather than the experimentally observed increase in *H*_ex_.

**Fig. 3.**
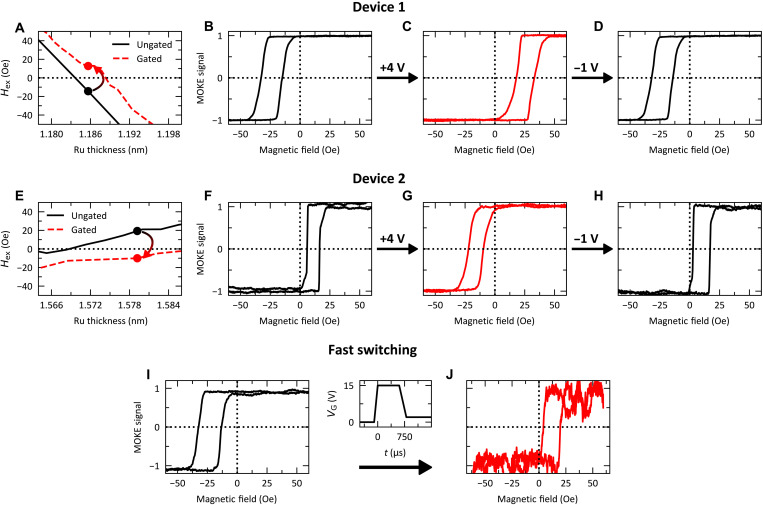
Magneto-ionic gating of the RKKY coupling. Exchange field as a function of ruthenium thickness near the nodes corresponding to a device that has FM coupling (**A**) and AFM coupling (**E**). (**B** to **D** and **F** to **H**) Polar MOKE minor hysteresis loops of the ungated state (**B** and **F**), after an applied gated voltage of +4 V for 30 s (**C** and **G**), and after the applied gate voltage is set to −1 V for 30 s, the recovered state (**D** and **H**). (**I** and **J**) Submillisecond switching using a gate voltage of +15 V for 750 μs.

The phase shift allows for switching of the RKKY coupling from FM to AFM and vice versa for an interlayer thickness near the RKKY zero crossings. To demonstrate this, devices near the crossing points were chosen, shown in Fig. 3 (A and E). Initially, device 1 has FM coupling, *H*_ex_ < 0, shown in the minor hysteresis loop in Fig. 3B. Under *V*_g_ = +4 V for 30 s, the sign of *H*_ex_ switches from negative to positive, shown in Fig. 3C, indicating that the RKKY coupling has switched from FM to AFM. Then, using *V*_g_ = −1 V for 30 s, the original state is fully recovered, shown in Fig. 3D. In device 2, where the Ru interlayer is thicker, we demonstrate the inverse. The voltage-induced changes under the same *V*_g_ are shown in Fig. 3 (F to H). The switching is fully reversible for several cycles in both experiments and demonstrates full, dynamic control of the strength and sign of the RKKY coupling with a small bias voltage. The switching can also be submillisecond. By applying a +15 V square-wave pulse for 750 μs, the coupling changes from FM to AFM, shown in Fig. 3 (I and J), respectively. After the voltage pulse, the device was held at *V*_g_ = +2 V to prevent the spontaneous discharging of H when grounded. The dependence of the switching time on the gate voltage from +4 to +15 V is shown in fig S3. When these devices are set to open circuit, i.e., *V*_g_ is removed, and there is a low leakage current, the gated state is nonvolatile; however, when grounded (*V*_g_ = 0), the initial state spontaneously recovers because of the spontaneous unloading of H (*24*). To prevent this, a finite positive *V*_g_ can be applied. A control experiment was performed with *V*_g_ = +2 V for 5 min to ensure that there was no change in the magnetic properties, shown in fig. S4. A recent demonstration of voltage-induced switching using the same solid-state proton pump platform has shown that magneto-ionic switching times can be as low as 50 μs (*41*); with further optimization, similar time scales for these devices can likely be achieved.

### 180° field-free switching

Voltage-controlled RKKY coupling further opens the door for manipulation of spintronic devices. We show this through 180° field-free switching of a free layer induced by changing *H*_ex_ > 2H_c_, thus transforming the remanent magnetization state to the opposite direction. H injection causes a change in reflectivity (*40*), which obscures the step change in the MOKE signal during *V*_g_-triggered magnetization switching. We therefore demonstrate *V*_g_-induced switching by measuring the free layer orientation before and after *V*_g_ application using a pulsed magnetic field interrogation sequence. The sequence consists of three pulses: a set pulse (1200 Oe for 200 μs), a bipolar interrogation pulse (BIP) (± 120 Oe for 2 s each), and a recovery interrogation pulse (−120 Oe for 2 s). The interrogation pulses were only large enough to switch the magnetization of the free layer, while the set pulse was large enough to switch both layers.

**Fig. 4.**
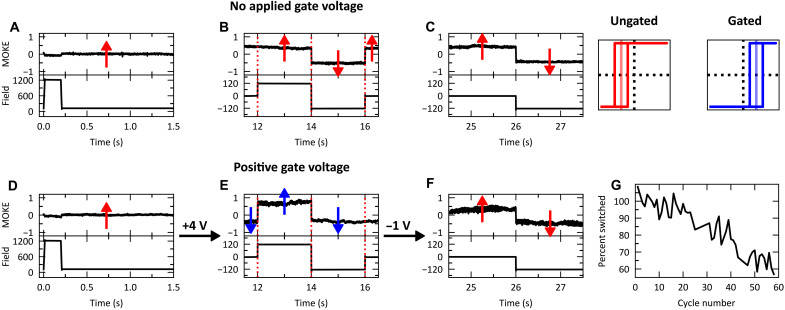
Voltage-modulated field-free switching. Using the device shown in Fig. 3 (A to D), the raw MOKE signal as a function of time is shown for ungated (**A** to **C**) and gated (**D** to **F**) states. The applied field waveform consisted of a set pulse of 1200 Oe for 200 μs (**A **and **D**), a BIP of ±120 Oe for 2 s each (**B** and **E**), and a recovery interrogation pulse of −120 Oe for 2 s (**C** and **F**). (**G**) Percent switched of the free layer for 60 cycles extracted using the ratio of the MOKE signal at *t* = 12 s over *t* = 14 s.

The experiment, shown in Fig. 4, uses device 1, which has *H*_ex_ < 0 in the ungated state; thus, the magnetization will switch when the field is swept from 0 to −120 Oe and generate a step feature in the MOKE signal. Initially, both layers are saturated up (Fig. 4A); therefore, during the positive field portion of the BIP (*t* = 12 s to *t* = 14 s), there is no change in the MOKE signal. However, during the remaining negative field portion of the BIP, the free layer switches to the down state, reaches negative saturation, and switches back to the up state as the field returns to zero, corresponding to the two steps in the MOKE signal at *t* = 14 s and *t* = 16 s, respectively (Fig. 4B).

The recovery interrogation pulse (Fig. 4C) therefore shows the same switching as at *t* = 14 s. The experiment was then repeated with *V*_g_ = +4 V between *t* = 3 s and *t* = 17 s. First, the device was saturated using the set pulse (Fig. 4D), and then, at *t* = 3 s and zero field, *V*_g_ = +4 V was applied. While *V*_g_ was applied, *H*_ex_ changes sign. Now, contrary to the previous experiment, two steps appear in the MOKE signal at *t* = 12 s and *t* = 14 s during the positive field portion of the BIP (Fig. 4E). This is only possible if, before the field, the magnetization of the free layer switched from the up state to the down state. Therefore, *V*_g_ alone induced 180° switching of the free layer through modification of the RKKY coupling. The negative field of the BIP, therefore, does not generate a change in the MOKE signal at *t* = 16 s. After the BIP, at *t* = 17 s and zero field, *V*_g_ is set to −1 V. This allows the RKKY coupling to return to its original state and leads to an additional field-free switching event. This is verified using the recovery interrogation pulse (Fig. 4F). Because there is a step in the MOKE signal during the negative field recovery pulse, the free layer must have been in the up state before the application of the field. Hence, the free layer magnetization switched from down to up.

The voltage-induced field-free switching is fully reversible and can be cycled several times without degradation (Fig. 4G). The percent switched is estimated using the ratio of the relative change in the MOKE signal at *t* = 12 s over *t* = 14 s. After approximately 20 cycles, the gating leads to a smaller change in *H*_ex_ for the same amount of voltage and time, leading to a decay in the estimated switching percentage. However, this decay can be accounted for by a decrease in the zero-field remanence, indicating that the switching process itself is quite reversible, as shown in the minor hysteresis loops of the free layer before and after several cycles (fig. S5). We believe that the degradation in the percent switched is due to a buildup of H in the Co/Pd hard layer leading to a reduced PMA. This can be seen in the shearing of the high-field switching events in the major loop after several cycles (fig. S6).

## DISCUSSION

We have shown that magneto-ionic gating of hydrogen can lead to an appreciable change in the RKKY coupling between magnetic multilayers in a solid-state heterostructure. This voltage-controlled manipulation is fully reversible, submillisecond, and can be cycled several times. We have shown that this can change the RKKY coupling of the multilayers from AFM to FM and vice versa. Moreover, by engineering multilayers to consist of a high coercivity (or pinned) fixed layer and low coercivity free layer, 180° field-free deterministic switching can be achieved using materials compatible with conventional magnetic tunnel junction processing conditions. We infer that the origin of this effect is the modification of the Fermi surface at the interface between the magnetic multilayers and the interlayer. We anticipate that this approach can be transferred to other materials and systems given the past work on hydrogen modulation of Fe/Nb (*44*), Fe/V (*45*), and GdCo (*41*). These results establish a dynamic, solid-state method of controlling a fundamental exchange interaction and show the potential for magneto-ionic control of spin-based memory devices and AFM spintronics.

## MATERIALS AND METHODS

### Sample preparation

All samples were grown using DC magnetron sputtering at room temperature and at a background pressure < 1 × 10^−7^ torr. The heterostructure was Ta(3)/Pt(2.5)/[Co(0.27)/Pt(1.2)]_2_/Co(0.27)/Ru(0.4–1.7)/Co(0.3)/[Pd(1.2)/Co(0.3)]_5_/Pd(6)/GdO*_x_*(26)/Au(8) (in nanometers) and grown on 50-nm wet thermally oxidized silicon (100) substrates (University Wafer). Au was grown under 3.5 mtorr Ar, while the rest of the metal layers were grown under 3 mtorr Ar. The GdO*_x_* layer was deposited using radio frequency sputtering with 3 mtorr Ar and 0.7 mtorr O_2_. The wedge growth of the Ru layer was achieved using a spatially varying mask over a distance of about 20 mm. The heterostructure was grown with a vacuum break after the first Pt layer, Ru wedge, and GdO*_x_* layer. The continuous films were then shadow-masked to pattern the Au electrodes used in the MOKE measurements. They had an approximate size of 550 μm by 100 μm (long direction parallel to the gradient of the wedge direction) and were separated by approximately 250 μm parallel and perpendicular to the wedge direction. The samples in Fig. 2 (A and B) were grown first and separated. The thicknesses of the Co, Pt, and Pd, along with the number of repeat units, were chosen to maintain strong PMA under hydrogen loading. The thickness of the Ru interlayer was determined by first patterning a substrate with lines and sputtering a calibration film. The height of those lines was measured as a function of position using an atomic force microscope. The data were then fit, and this fit was used to determine the nominal thickness of the Ru interlayer as a function of position on the sample.

### Polar MOKE measurements

Polar MOKE measurements were performed using a 1-mW laser with a wavelength of 660 nm focused to a spot size of about 10 μm. The polar geometry enables the measurement of the out-of-plane magnetization. A CuBe probe was used to make electrical contact with the top Au electrodes. A second CuBe probe was used to make electrical contact with the bottom electrode, which was the Pd capping layer. Before the deposition of the GdO*_x_* layer, a small section of the Pd layer was covered at the sample edge for this purpose. The laser spot was positioned on the electrode during the MOKE measurements and during gating. The measurements along the wedge were done by rastering the sample underneath the laser using an XY direct-drive linear stage with 50 nm resolution (Aerotech ALS130H). For Figs. 1B and 2E, data points were taken every 100 μm along the wedge at various positions on 39 individual electrodes. All minor hysteresis loops were collected after first saturating the sample in the up direction and sweeping the field with a low enough magnitude to ensure that the hard layer would not switch.

### Cross-sectional HR-TEM

TEM cross section lift-out lamellas of the layer stack were prepared by focused ion beam milling. Bright-field TEM and HR-TEM were performed on these lamellas with an aberration-corrected Titan3 80-300 TEM instrument (Thermo Fisher Scientific) providing a resolution of 0.08 nm. The TEM micrographs were acquired at an acceleration voltage of 300 kV and recorded with a 2k by 2k slow-scan charge-coupled device camera (Gatan UltraScan 1000). To conduct qualitative elemental mapping, we carried out energy loss–filtered TEM (three-window method) using a postcolumn Gatan imaging filter (GIF Tridiem, Gatan Inc.). For the Gd mapping, the N_4,5_ edge at 141 eV was used; for the Pd and Pt mapping, the N_2,3_ and O_3_ edges at 51 and 52 eV (Pd and Pt not distinguishable here); for the Ru mapping, the M_4_ and M_5_ edges at 284 and 279 eV; for the Co mapping, the L_2_ and L_3_ edges at 779 and 794 eV; and for the O mapping, the K edge at 532 eV.

## References

[1] I. Dzyaloshinsky,A thermodynamic theory of “weak” ferromagnetism of antiferromagnetics. J. Phys. Chem. Solid 4,241–255 (1958).

[2] T. Moriya,Anisotropic superexchange interaction and weak ferromagnetism. Phys. Rev. 120,91–98 (1960).

[3] M. A. Ruderman, C. Kittel,Indirect exchange coupling of nuclear magnetic moments by conduction electrons. Phys. Rev. 96,99–102 (1954).

[4] T. Kasuya,A theory of metallic ferro- and antiferromagnetism on Zener’s model. Prog. Theor. Phys. 16,45–57 (1956).

[5] K. Yosida,Magnetic properties of Cu-Mn alloys. Phys. Rev. 106,893–898 (1957).

[6] S. S. P. Parkin,Systematic variation of the strength and oscillation period of indirect magnetic exchange coupling through the 3d, 4d, and 5d transition metals. Phys. Rev. Lett. 67,3598–3601 (1991). 10044776 10.1103/PhysRevLett.67.3598

[7] S. Parkin, X. Jiang, C. Kaiser, A. Panchula, K. Roche, M. Samant,Magnetically engineered spintronic sensors and memory. Proc. IEEE 91,661–680 (2003).

[8] E. V. Gomonay, V. M. Loktev,Spintronics of antiferromagnetic systems (Review article). Low Temp. Phys. 40,17–35 (2014).

[9] T. Jungwirth, X. Marti, P. Wadley, J. Wunderlich,Antiferromagnetic spintronics. Nat. Nanotechnol. 11,231–241 (2016). 26936817 10.1038/nnano.2016.18

[10] V. Baltz, A. Manchon, M. Tsoi, T. Moriyama, T. Ono, Y. Tserkovnyak,Antiferromagnetic spintronics. Rev. Mod. Phys. 90,015005 (2018).

[11] L. Šmejkal, Y. Mokrousov, B. Yan, A. H. MacDonald,Topological antiferromagnetic spintronics. Nat. Phys. 14,242–251 (2018).

[12] S.-H. Yang, K.-S. Ryu, S. Parkin,Domain-wall velocities of up to 750 m^s-1^ driven by exchange-coupling torque in synthetic antiferromagnets. Nat. Nanotechnol. 10,221–226 (2015). 25705867 10.1038/nnano.2014.324

[13] O. Gueckstock, L. Nádvorník, M. Gradhand, T. S. Seifert, G. Bierhance, R. Rouzegar, M. Wolf, M. Vafaee, J. Cramer, M. A. Syskaki, G. Woltersdorf, I. Mertig, G. Jakob, M. Kläui, T. Kampfrath,Terahertz spin-to-charge conversion by interfacial skew scattering in metallic bilayers. Adv. Mater. 33,2006281 (2021). 10.1002/adma.202006281 PMC11469024 33506577

[14] S. S. P. Parkin, M. Hayashi, L. Thomas,Magnetic domain-wall racetrack memory. Science 320,190–194 (2008). 18403702 10.1126/science.1145799

[15] W. Koshibae, N. Nagaosa,Theory of skyrmions in bilayer systems. Sci. Rep. 7,42645 (2017). 28198436 10.1038/srep42645 PMC5309827

[16] W. Legrand, D. Maccariello, F. Ajejas, S. Collin, A. Vecchiola, K. Bouzehouane, N. Reyren, V. Cros, A. Fert,Room-temperature stabilization of antiferromagnetic skyrmions in synthetic antiferromagnets. Nat. Mater. 19,34–42 (2020). 31477905 10.1038/s41563-019-0468-3

[17] X. Zhang, Y. Zhou, M. Ezawa,Magnetic bilayer-skyrmions without skyrmion Hall effect. Nat. Commun. 7,10293 (2016). 26782905 10.1038/ncomms10293 PMC4735649

[18] J. Barker, O. A. Tretiakov,Static and dynamical properties of antiferromagnetic skyrmions in the presence of applied current and temperature. Phys. Rev. Lett. 116,147203 (2016). 27104724 10.1103/PhysRevLett.116.147203

[19] T. Dohi, S. DuttaGupta, S. Fukami, H. Ohno,Formation and current-induced motion of synthetic antiferromagnetic skyrmion bubbles. Nat. Commun. 10,5153 (2019). 31727895 10.1038/s41467-019-13182-6 PMC6856122

[20] P. Wadley, B. Howells, J. Železný, C. Andrews, V. Hills, R. P. Campion, V. Novák, K. Olejník, F. Maccherozzi, S. S. Dhesi, S. Y. Martin, T. Wagner, J. Wunderlich, F. Freimuth, Y. Mokrousov, J. Kuneš, J. S. Chauhan, M. J. Grzybowski, A. W. Rushforth, K. W. Edmonds, B. L. Gallagher, T. Jungwirth,Electrical switching of an antiferromagnet. Science 351,587–590 (2016). 26841431 10.1126/science.aab1031

[21] T. Xu, H.-A. Zhou, Y. Dong, Q. Zhang, M. Che, L. Liu, Z. Wu, Z. Guan, L. Yang, W. Jiang,Fully compensated synthetic antiferromagnets with pronounced anomalous hall and magneto-optical responses. Phys. Rev. Applied 16,044056 (2021).

[22] S. Bhatti, R. Sbiaa, A. Hirohata, H. Ohno, S. Fukami, S. N. Piramanayagam,Spintronics based random access memory: A review. Mater. Today 20,530–548 (2017).

[23] U. Bauer, L. Yao, A. J. Tan, P. Agrawal, S. Emori, H. L. Tuller, S. van Dijken, G. S. D. Beach,Magneto-ionic control of interfacial magnetism. Nat. Mater. 14,174–181 (2015). 25401920 10.1038/nmat4134

[24] A. J. Tan, M. Huang, C. O. Avci, F. Büttner, M. Mann, W. Hu, C. Mazzoli, S. Wilkins, H. L. Tuller, G. S. D. Beach,Magneto-ionic control of magnetism using a solid-state proton pump. Nat. Mater. 18,35–41 (2019). 30420669 10.1038/s41563-018-0211-5

[25] K.-Y. Lee, S. Jo, A. J. Tan, M. Huang, D. Choi, J. H. Park, H.-I. Ji, J.-W. Son, J. Chang, G. S. D. Beach, S. Woo,Fast magneto-ionic switching of interface anisotropy using yttria-stabilized zirconia gate oxide. Nano Lett. 20,3435–3441 (2020). 32343588 10.1021/acs.nanolett.0c00340

[26] M. Ameziane, R. Mansell, V. Havu, P. Rinke, S. van Dijken,Lithium-ion battery technology for voltage control of perpendicular magnetization. Adv. Funct. Mater. 32,2113118 (2022).

[27] J. Zehner, R. Huhnstock, S. Oswald, U. Wolff, I. Soldatov, A. Ehresmann, K. Nielsch, D. Holzinger, K. Leistner,Nonvolatile electric control of exchange bias by a redox transformation of the ferromagnetic layer. Adv. Electron. Mater. 5,1900296 (2019).

[28] J. Zehner, D. Wolf, M. U. Hasan, M. Huang, D. Bono, K. Nielsch, K. Leistner, G. S. D. Beach,Magnetoionic control of perpendicular exchange bias. Phys. Rev. Mater. 5,L061401 (2021).

[29] P. D. Murray, C. J. Jensen, A. Quintana, J. Zhang, X. Zhang, A. J. Grutter, B. J. Kirby, K. Liu,Electrically enhanced exchange bias via solid-state magneto-ionics. ACS Appl. Mater. Interfaces 13,38916–38922 (2021). 34347431 10.1021/acsami.1c11126

[30] Q. Yang, L. Wang, Z. Zhou, L. Wang, Y. Zhang, S. Zhao, G. Dong, Y. Cheng, T. Min, Z. Hu, W. Chen, K. Xia, M. Liu,Ionic liquid gating control of RKKY interaction in FeCoB/Ru/FeCoB and (Pt/Co)_2_/Ru/(Co/Pt)_2_ multilayers. Nat. Commun. 9,991 (2018). 29515180 10.1038/s41467-018-03356-z PMC5841336

[31] Q. Yang, Z. Zhou, L. Wang, H. Zhang, Y. Cheng, Z. Hu, B. Peng, M. Liu,Ionic gel modulation of RKKY interactions in synthetic anti-ferromagnetic nanostructures for low power wearable spintronic devices. Adv. Mater. 30,1800449 (2018). 10.1002/adma.201800449 29663532

[32] L. Herrera Diez, Y. T. Liu, D. A. Gilbert, M. Belmeguenai, J. Vogel, S. Pizzini, E. Martinez, A. Lamperti, J. B. Mohammedi, A. Laborieux, Y. Roussigné, A. J. Grutter, E. Arenholtz, P. Quarterman, B. Maranville, S. Ono, M. S. E. Hadri, R. Tolley, E. E. Fullerton, L. Sanchez-Tejerina, A. Stashkevich, S. M. Chérif, A. D. Kent, D. Querlioz, J. Langer, B. Ocker, D. Ravelosona,Non-volatile ionic modification of the dzyaloshinskii moriya interaction. Phys. Rev. Appl. 12,34005 (2019).

[33] S. S. P. Parkin, N. More, K. P. Roche,Oscillations in exchange coupling and magnetoresistance in metallic superlattice structures: Co/Ru, Co/Cr, and Fe/Cr. Phys. Rev. Lett. 64,2304–2307 (1990). 10041640 10.1103/PhysRevLett.64.2304

[34] S. S. P. Parkin, R. Bhadra, K. P. Roche,Oscillatory magnetic exchange coupling through thin copper layers. Phys. Rev. Lett. 66,2152–2155 (1991). 10043404 10.1103/PhysRevLett.66.2152

[35] B. Dieny, J. P. Gavigan, J. P. Rebouillat,Magnetisation processes, hysteresis and finite-size effects in model multilayer systems of cubic or uniaxial anisotropy with antiferromagnetic coupling between adjacent ferromagnetic layers. J. Phys. Condens. Matter 2,159–185 (1990).

[36] P. F. Carcia,Perpendicular magnetic anisotropy in Pd/Co and Pt/Co thin-film layered structures. J. Appl. Phys. 63,5066–5073 (1988).

[37] S. A. Steward, “Review of Hydrogen Isotope Permeability Through Materials” (Lawrence Livermore National Laboratory, 1983).

[38] J. Rossmeisl, A. Logadottir, J. Nørskov,Electrolysis of water on (oxidized) metal surfaces. Chem. Phys. 319,178–184 (2005).

[39] A. J. Tan, M. Huang, S. Sheffels, F. Büttner, S. Kim, A. H. Hunt, I. Waluyo, H. L. Tuller, G. S. D. Beach,Hydration of gadolinium oxide (Gd O*_x_*) and its effect on voltage-induced Co oxidation in a Pt/Co/Gd O*_x_*/Au heterostructure. Phys. Rev. Mater. 3,064408 (2019).

[40] M. Huang, A. Jun Tan, F. Büttner, H. Liu, Q. Ruan, W. Hu, C. Mazzoli, S. Wilkins, C. Duan, J. K. W. Yang, G. S. D. Beach,Voltage-gated optics and plasmonics enabled by solid-state proton pumping. Nat. Commun. 10,5030 (2019). 31695041 10.1038/s41467-019-13131-3 PMC6834670

[41] M. Huang, M. U. Hasan, K. Klyukin, D. Zhang, D. Lyu, P. Gargiani, M. Valvidares, S. Sheffels, A. Churikova, F. Büttner, J. Zehner, L. Caretta, K.-Y. Lee, J. Chang, J. P. Wang, K. Leistner, B. Yildiz, G. S. D. Beach,Voltage control of ferrimagnetic order and voltage-assisted writing of ferrimagnetic spin textures. Nat. Nanotechnol. 16,981–988 (2021). 34326528 10.1038/s41565-021-00940-1

[42] A. O. Leon, J. d’Albuquerque e Castro, J. C. Retamal, A. B. Cahaya, D. Altbir,Manipulation of the RKKY exchange by voltages. Physical Review B 100,014403 (2019).

[43] J. d’Albuquerque e Castro, D. Altbir, A. O. Leon, J. C. Retamal,Phase-shift control of the exchange coupling between magnetic impurities. Nanotechnology 31,355002 (2020). 32396875 10.1088/1361-6528/ab9259

[44] F. Klose, C. Rehm, D. Nagengast, H. Maletta, A. Weidinger,Continuous and reversible change of the magnetic coupling in an Fe/Nb multilayer induced by hydrogen charging. Phys. Rev. Lett. 78,1150–1153 (1997).

[45] B. Hjörvarsson, J. A. Dura, P. Isberg, T. Watanabe, T. J. Udovic, G. Andersson, C. F. Majkrzak,Reversible tuning of the magnetic exchange coupling in Fe/V (001) superlattices using hydrogen. Phys. Rev. Lett. 79,901–904 (1997).

