# Supplementary material for: Voltage control of magnetic order in RKKY coupled multilayers
Source: Sci Adv. 2023 Jan 4;9(1):eadd0548. doi: 10.1126/sciadv.add0548 (PMC9812395; doi:10.1126/sciadv.add0548)
Supplement: Supplementary file 1 — Supplementary Text Figs. S1 to S6 [file sciadv.add0548_sm.pdf]

Science Advances
AAAS

Supplementary Materials for

Voltage control of magnetic order in RKKY coupled multilayers

Alexander E. Kossak *et al.*

Corresponding author: Alexander E. Kossak, akossak@mit.edu

Sci. Adv. **9**, eadd0548 (2022)
DOI: 10.1126/sciadv.add0548

This PDF file includes:

Supplementary Text
Figs. S1 to S6

Supplementary Text

Hydrogen penetration of buried magnetic layers

Previous demonstrations of hydrogen modulation of GdCo (41) indicated that GdCo is an ideal platform to test the penetration depth of hydrogen. If the GdCo is grown as Gd-rich with perpendicular magnetic anisotropy, then it is possible to inject enough hydrogen into the system to change the dominant sublattice and switch the MOKE polarity of the hysteresis loop. As opposed to monitoring changes in the coercivity, the change in MOKE polarity is a clear indication that hydrogen has reached the buried magnetic layer. In order to demonstrate that hydrogen could penetrate the relatively thick Co/Pd multilayer and the Ru interlayer, we fabricated the heterostructure: Ta(3)/Pt(4)/GdCo(8)/Ru(2)/Pd(13.8)/GdO_x(26)/Au(8), where the GdCo layer is on the bottom, serving as a hydrogen detection layer and with the same thickness of the Co/Pd multilayer on top, however, with the Co omitted. The Co was omitted so the MOKE switching events could be unambiguously attributed to the buried GdCo layer. The results shown in Fig. S2 indicate that hydrogen can indeed penetrate several nanometers of Ru and Pd and into the GdCo layer. Moreover, it can also be easily removed as the recovered state returns to Gd-dominated GdCo.

Sub-millisecond holding voltage

Using a neighboring device with respect to the one used in Fig. 3I-J, we tested the stability of the exchange coupling under an applied bias of +2 V. This bias was used in the experiments shown in Fig. 3 to prevent the spontaneous discharging of hydrogen when the device is grounded (Vg = 0 V). Even after 5 minutes, it’s clear that there is no change in the exchange field, and therefore the RKKY coupling.

Field-free switching cycles

As shown in the main text and Fig. 4, the voltage-induced field-free switching is fully reversible and can be cycled several times without degradation. After approximately 20 cycles, the positive applied bias leads to a smaller change in the Co/Pt soft layer for the same amount of applied voltage and time. The estimated percent switched (the relative change in raw MOKE signal at t = 12 s over t = 14 s) therefore decreases. This is seen in the gated minor hysteresis loops of the free layer in the first cycle (Fig. S5A) versus the 140th cycles (Fig. S5B). The ungated and recovered states have no significant change and it is possible to re-induced field-free switching with a longer or higher applied voltage bias. We believe the origin of the degradation can be attributed to the Co/Pd hard layer. As can be seen in the major vs minor hysteresis loop of a separate device in Fig. S6A-B; the minor loop (Co/Pt layer) remains square under higher gate voltages than used previously, however, in the major loop, there is shearing of the switching events at high fields. The layer switching at high field is the hard layer. Indicating that over repeated cycles there is likely not enough time for the hydrogen to completely unload from the Co/Pd multilayer and the buildup of hydrogen in the hard layer could be reducing the perpendicular magnetic anisotropy. We believe, with further optimization of the hard, reference layer, the device should be cyclable for significantly more cycles. In addition, Fig. 4G shows some cycles have a percent switched in excess of 100 %. This can be attributed to a spurious change in the reflected laser intensity due to the change in refractive index of GdO_x when loaded with hydrogen (40).

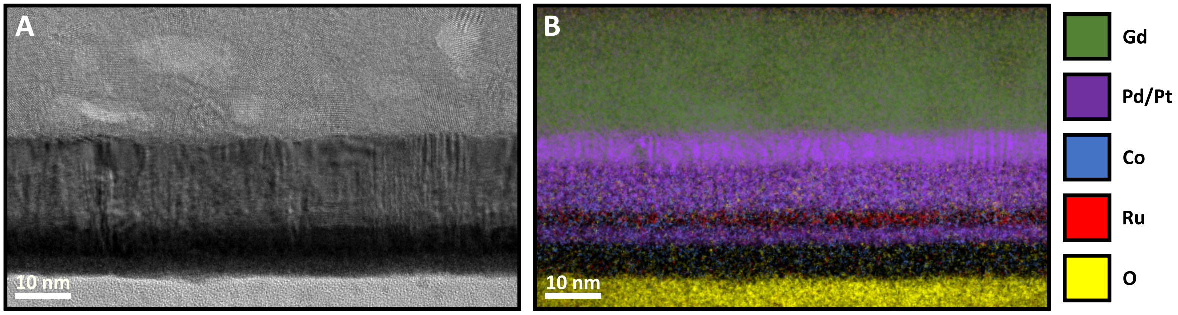

Fig. S1: Heterostructure interface quality | **A,** Cross-sectional high-resolution transmission electron microscopy (HR-TEM) of Ta(3)/Pt(2.5)/[Co(0.27)/Pt(1.2)]$_2$/Co(0.27)/Ru(1.7)/Co(0.3)/[Pd(1.2)/Co(0.3)]$_5$/Pd(6)/GdO$_x$(26)/Au(8) and **B,** the corresponding elemental mapping for Gd, Pd/Pt (indistinguishable), Co, Ru, and O.

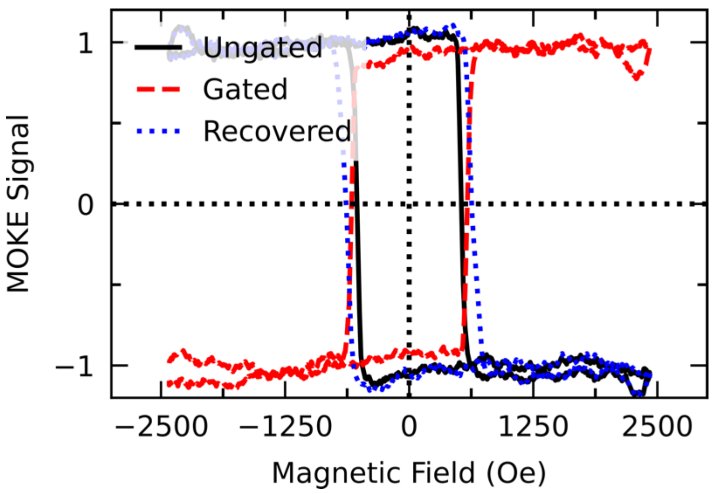

Fig. S2: Magneto-ionic gating of buried GdCo | Polar MOKE hysteresis loops of Ta(3)/Pt(4)/GdCo(8)/Ru(2)/Pd(13.8)/GdO_x(26)/Au(8) are given for the ungated, gated, and recovered states. The gated state was achieved using a bias of +2 V for 30 s and the recovered state was achieved using a bias of -1V for 30 s.

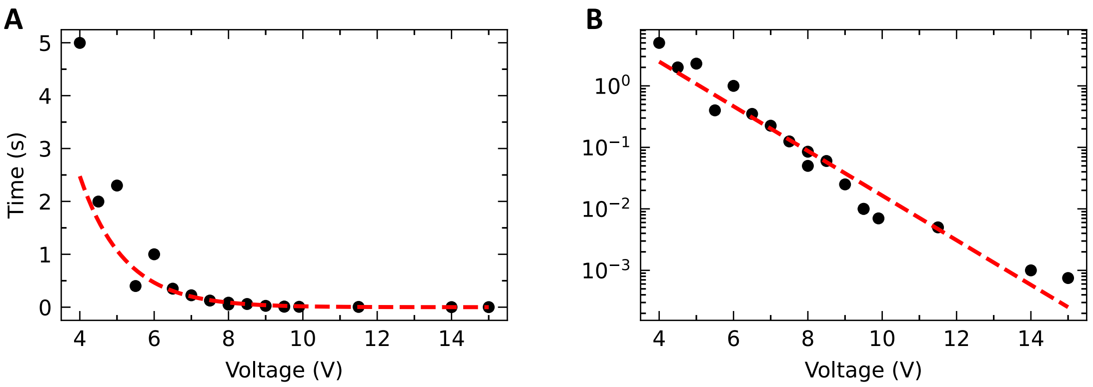

Fig. S3: Switching time for several gate voltages | Pulse time needed at a given voltage in order to induce switching of the RKKY coupling as performed in Fig. 3. The data was aggregated for two samples and in shown with both a linear (**A**) and log (**B**) scale.

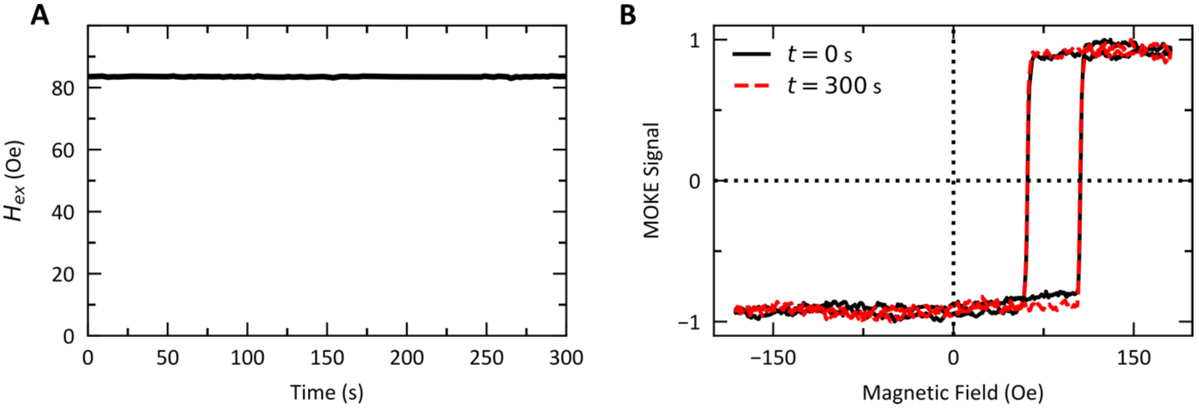

Fig. S4: Magneto-ionic gating at +2 V for 300 s | A, Exchange field as a function of time extracted from the polar MOKE minor hysteresis loops. **B**, Polar MOKE minor hysteresis loops at t = 0 s (black, solid) and t = 300 s (red, dashed).

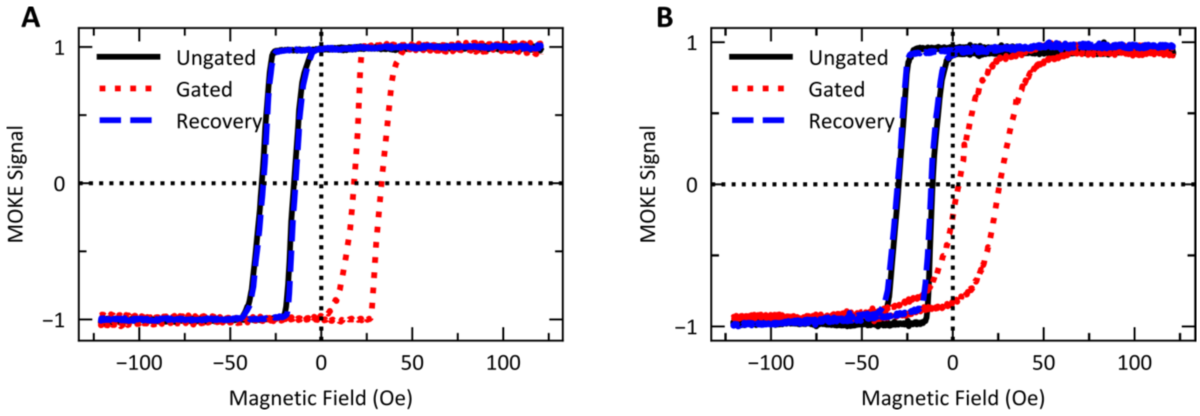

Fig. S5: Cycling stability of field-free switching | A, First cycle and **B**, 140th cycle using the device shown in Figs. 3A-D and Figs. 4A-G. The minor hysteresis loops of the multilayer heterostructure are given for the ungated, gated, and recovered state. A bias of +4 V for 14 s is applied to reach the gated state and then a bias of -1 V for 10 s to reach the recovered state.

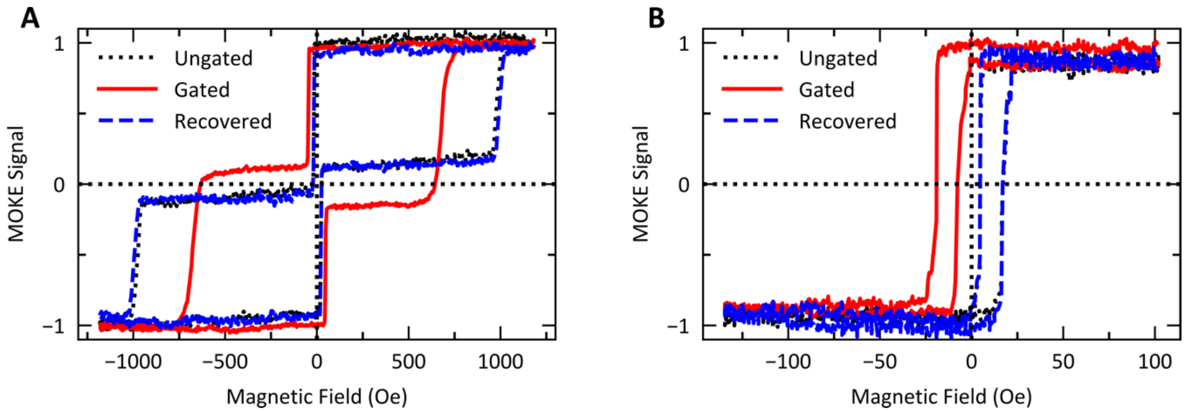

Fig. S6: Heterostructure stability under cycling | **A,** Major and **B,** minor polar MOKE minor hysteresis loops after cycling with an applied bias of +4.5 V for 30 s.
